# The Role of Emotional Intelligence and Sociocultural Adjustment on Migrants’ Self-reported Mental Well-Being in Spain: A 14 Month Follow-Up Study

**DOI:** 10.3390/ijerph17041206

**Published:** 2020-02-13

**Authors:** José Luis González-Castro, Silvia Ubillos Landa, Alicia Puente Martínez, Maria Vera Perea

**Affiliations:** 1 Educational Science Department, University of Burgos (Spain), C/Villadiego 1, 09001 Burgos, Spain; 2 Health Science Department, University of Burgos (Spain), P Comendadores s/n, 09001 Burgos, Spain; subillos@ubu.es; 3 Social Psychology and Methodology Department, Basque Country University, Avda. Tolosa, 70, 20018 Gipuzkoa, Spain; apuentema@gmail.com; 4 Department of Education and Social Psychology, Universidad Pablo de Olavide, Ctra. de Utrera, km. 1, 41013 Sevilla, Spain; mverper1@upo.es

**Keywords:** migration, mental well-being, emotional intelligence, public health, follow-up study, sociocultural adjustment

## Abstract

The analysis of mental and psychological health is a relevant public issue in modern societies. Migration is a process that may have a lasting impact on a person’s mental well-being. In this study, perceived health, emotional intelligence, sociocultural adjustment and the participants’ perceived general situation, not only economical, were analyzed to attest their impact on psychological distress as a measure of mental well-being. Sixty-three migrants from Romania and Ecuador were contacted twice during a 14 month period in a middle-sized Spanish city. Attrition analyses show no significant differences in perceived psychological distress between those who participated only one time or who participated in both waves. Less psychological distress is related to less attention to one’s feelings and higher mood repair in both data waves. Stronger behavioral adjustment is also linked to less distress. Less distress in time 1 led to better perceived health, sociocultural adjustment and a perception of a better general situation in Spain in comparison to their home country in time 2. In general, more attention to negative feelings triggered more perceived psychological distress, whereas mood repair elicited less psychological distress, in time 2. The relevance of understanding the impact of emotional intelligence to health promotion programs with migrants is discussed.

## 1. Introduction

International migrants represent approximately 258 million people in 2017, roughly 3.4% of the world population [1]. Although their social, political and economic relevance is noteworthy, at the same time migrants are often engaged in 3-D jobs (dirty, dangerous and demanding), and work for less pay, in worse conditions or take greater risks in the job without adequate training [2]. The number of migrants enduring human rights violations, discrimination, persecution or abuse has increased in the last decade [3].

In this context, the impact of migration on a person’s social and psychological well-being and health, and its consequences for public health policies, is an important research and social issue. Although this study will focus specifically on the mental well-being of migrants and the impact that their experience and relation with a new environment may have on this issue, health problems affect large numbers of people in any society and must be treated as a global problem. Different authorities have estimated the prevalence of mental disorders in the European region and the US in a range of 12–19% of the population [4,5]. Migrating to a new country and the personal, social and contextual changes it implies may be a novel experience enhancing a person’s potential and improving one’s economic or social well-being, but it can also be a stressful life experience that could lead to acculturative stress. Acculturation can be broadly defined as a series of cultural changes (practices, values or identification processes) experienced through intercultural contact. The emergence of stressors or protective factors specific to these cultural transitions may produce psychological distress if the individual lacks sufficient resources, or the host culture does not grant resources, to cope with the new situation [6]. As Archuleta and Lakwhani [7] mention, living in a new cultural environment requires possessing a series of environmental mastery skills (e.g., code switching, accommodating one’s speech, language learning, regulating emotion/mood, participating physically and mentally in activities outside one’s own environment) that reflect a person’s ability to manage complex situations and identify resources within the environment that help achieve his or her needs.

Migrants face larger possible distress prevalence than native-born populations due to individual, social, and economic factors that may appear during the acculturation process [8]. This type of stress may have various mental well-being consequences, including affective disorders, anxiety, and adjustment difficulties. A meta-analysis [9] conducted including 21 studies with first- and second-generation immigrants found that there was an increased risk for schizophrenia and other related severe mental disorders in both groups of participants.

Nevertheless, a substantial number of empirical studies have also revealed non-significant, positive or mixed effects of acculturation on mental health and well-being [10,11]. Ronda-Pérez, Martínez, Reid and Agudelo-Suárez [12] reported a healthy immigrant worker effect, showing that new arrivals in Spain from Ecuador and Colombia had a lower incidence of common mental disorders than Spanish-born workers, or immigrants who had lived in Spain for more than 15 years. Rudmin [13] showed that acculturation did not necessarily elicit psychological distress, and that the consequences of acculturation needed to be understood, analysing contextual and environmental variables that could act as protective or risk factors in this process. Scheffran, Marmer and Sow [14] point out that migrants must be seen as active social agents who shape their lives under changing environmental conditions, and that human migration is an historical response to poverty, dissatisfaction and environmental change. It does not necessarily imply negative consequences for the person who migrates or for the host culture.

Adjusting to a new cultural context is a process that allows people to settle and function both physically and mentally within a new environment, be it out of choice or need [15]. The process of acculturation observed in immigrants is part of an adjustment to the values and norms of a new society, and can imply both maintaining and/or losing some norms from the society of origin [16]. Sociocultural adjustment or adaptation is a social learning process and refers to acquiring and understanding a series of social skills and knowing how to manage different aspects of the host society. Ward and Kennedy [17] stated that sociocultural adaptation was composed of a bidimensional structure: a cognitive factor, pertaining to being acquainted with the views and values of locals, and a behavioral factor termed cultural learning, or how to deal with impersonal situations such as bureaucracy or getting along with unpleasant people. Nevertheless, simply analyzing cognitive or behavioral strengths neglects the role of emotional factors that can act as protective factors against mental health challenges. Schneeberger et al. [18] reported data showing how the emotional distress suffered by immigrants had a negative effect on their insomnia in comparison to non-immigrants.

Emotional acculturation is defined as a series of changes in emotional patterns as a consequence of an immigrant’s contact with a new culture [19]. Archuleta and Lakwani [7] posit that emotional regulation is necessary to master the environment and would therefore mediate the importance of depressive symptoms, acting as a protective factor. When analyzing emotions and their regulation, one of the most relevant variables currently researched is the study of Emotional Intelligence (EI). This concept has been defined as a subtype of intelligence that comprises the ability to perceive and integrate emotion in order to facilitate thought, understand and regulate emotions to promote personal growth (see [20] for more information). EI refers to an adequate interaction between emotion and cognition, allowing an individual to become adjusted to his or her environment [21]. A person’s level of Emotional Intelligence could help them better cope with the stress derived from challenges associated with facing novel or unforeseen situations due to the observed positive correlation between high EI and fewer stress symptoms [22]. Baudry, Grynberg, Dassonneville, Lelorain and Christophe [23] mention that people with higher EI are more likely to understand, regulate and use emotional information to cope with daily stressors (e.g., everyday work concerns, relations with others, discrimination) and thus have less problems within their environment and have better health.

In a meta-analysis conducted by Schutte, Malouff, Thorsteinsson, Bhullar, and Rooke [24], the correlation between EI and mental health was 0.29. Moreover, Baudry et al.’s [23] systematic review of the literature stresses that high scores in EI and its sub-dimensions are associated with better mental, physical and general health in clinical and non-clinical populations. Moreover, the analysis of the subdimensions of trait EI is more informative of the relation between emotion and health than the global score [23]. EI thus may be used as a plausible general predictor of mental health and well-being. Nevertheless, scarce research has been conducted on the relationship between EI and cross-cultural contexts [25,26]. Schmitz and Schmitz in Germany [27] and López-Zafra and El Ghoudani [28] in Spain showed that EI helped regulate acculturation and interpersonal relationships, reducing its negative impact and maintaining a level of emotional and social balance. EI may act as a buffer against acculturative stress, increasing socio-cultural adjustment and positive mental health [29]. An adjusted emotional expression, or high EI score, could reduce psychological stress and poor adaptation [30].

The three dimensions of emotional intelligence—(1) attention to feelings (thinking about or notice one’s feelings); (2) emotional clarity (identify and discriminate between feelings); and (3) mood repair (regulate moods and repair negative emotional experiences) measured using the Trait Meta Mood Scale (TMMS) [31]—play different roles in the study of mental health and well-being. Emotional clarity and emotional mood repair are negatively correlated with depression and anxiety whilst attention is positively related [23,25,32]. Greater repair is most strongly associated with better mental health, while more attention is most strongly related to psychological distress [23]. Gohm and Clore [33] found that whilst clarity was related to emotional mood repair and well-being, attention to emotions was related to neither. A high level of emotional attention towards negative affects was linked to high intensity and difficulty regulating emotional states. Schutte et al. [24] found that when strong attention to emotions was not compensated with adequate levels of clarity and mood repair there was a surge in ruminative thoughts and negative emotional states related to depression. Shulman and Hemenover [34] reported that attention to negative feelings was the strongest predictor of emotional distress, whilst mood repair and clarity predicted psychological well-being. In sum, although EI may act as a buffer in mental health, paying too much attention to one’s emotions could have a negative impact in one’s process of becoming involved with a new environment. This may be the case in the migrant population due to constraints imposed by learning to live within a new social, geographical and relational context. However, there are scarce studies in the literature which employ longitudinal or follow-up data to research the predictive impact of EI on health [23]. This is an even more acute problem in the study of emotion regulation and adjusting to migration due to the practical nonexistence of previous research within this domain.

Based on the abovementioned studies, the main objective of this research was to study how the analysis of the mental well-being of migrants may benefit from the inclusion of emotion-related variables. More specifically, we address the interaction of EI and socio-cultural adjustment and its impact on the mental well-being of migrants. Consequently, this study postulated that (1) EI and socio-cultural adjustment will have a direct impact on psychological distress. We also evaluated whether, over time, migrants high in emotion perception would be more influenced by stress than those low in perception, suggesting that (2) there will be a weaker link between acculturative stress and mental well-being among participants who focused relatively less on negative moods and emotions.

Finally, it is noted that while theories hypothesize that stress, acculturation and socio-cultural adjustment are a temporal and longitudinal process, few empirical studies have actually identified this relationship, and studies continue to be based heavily on cross-sectional survey designs. This methodological mismatch limits the potential to fully capture and understand the multifaceted, sequential and progressive nature of distress–psychosocial adjustment experienced by migrants. Thus, the analysis of such variables within longitudinal or follow-up studies can be a necessary empirical and theoretical addition to the existing literature.

## 2. Materials and Methods

### 2.1. Participants and Procedure

Participants were Romanian and Ecuadorian migrants living in Spain. The proportion of Romanian and Ecuadorian migrants represent one of the most relevant groups in Spain [35], and so these were deemed relevant research groups for an acculturation study. Members of the research team visited different Ecuador and Romanian migrant associations in a middle-sized Spanish city explaining the aim of the study and contacting those voluntarily willing to participate. Participants, who were mostly actively working migrants, completed the self-reported data. They were not asked about their legal and administrative status although all had entered Spain legally and mentioned that their reasons for migrating were to improve economically (economic migrants) or to reunite with their families. Although there was a Romanian translation of the questionnaire, all participants decided to answer the Spanish version with the assistance of a research team member. Answering the questionnaire took approximately 30 min and was completed in one individual face to face session with a trained member of the research team present to solve any problems the respondent could encounter.

Participants were contacted and informed of the general aim of the study (“analyze the experience of having migrated to Spain”). Although most participants answered the questionnaire at the head office of their association, others preferred to answer in other premises because of the problems reaching this head office (e.g., leaving work late, having to start work at a second job, taking care of family members). In these cases, a member of the research team met up with the participant at the location and time proposed by this person.

The sample in time 1 (T1) (*n* = 138) consisted of 81 Ecuadorians and 57 Romanians. After completion of this first study, participants were asked if they were willing to participate in a second study approximately 14 months later (range: 13–16 months). Those who agreed (96.4% of the original sample) instructed the researcher on how to contact them. The final study sample (*n* = 63) consisted of 21 participants (five men and 16 women) from Romania and 42 (15 men and 27 women) from Ecuador. Participants’ mean age was (in T1) 34 years (*SD* = 8.7). Mean length of stay in Spain in time 2 (T2) was 6.40 years (*SD* = 2.21). Although the sample size is not as large as desired, the lack of previous studies in the field of emotional intelligence and migration, or studies using longitudinal or follow up measures to analyze this relationship, may render it important to study this phenomena with the aim of guiding future research and setting clear theoretical and empirical bases for future work in public health settings [36].

Results yielded a dropout rate of 52.6%. This rate was expected because participants are labor migrants in areas such as construction or light industry in which geographic mobility is quite common. Most of the participants who did not answer the questionnaire in T2 were contacted but were not residing in the city anymore. The study’s protocol was approved by the university’s bioethical committee and complied with the ethical criteria on research with human beings (Declaration of Helsinki). All participants were instructed that they could abandon the research at any moment and that only the research team would have access to their results.

### 2.2. Instruments

*Perceived psychological distress* was measured with the 12-item General Health Questionnaire (GHQ-12) [37], adapted by Sánchez-López and Dresch [38]. A 4-point Likert-scale (0–3) response format was employed. Final scores were in the range 0–36. High scores imply higher perceived psychological distress.

Perceived health was measured with a single self-constructed item (“Overall, how would you describe your health at the present moment?” 1= very good, to 5 = very bad) based on previous studies conducted in Spain which showed adequate discrimination effects [39].

Emotional intelligence. Measured using 24 items of the widely used Trait Meta-Mood Scale (TMMS) [31], adapted by Fernández-Berrocal, Extremera, and Ramos [40]. This scale is a self-reporting questionnaire about one’s own emotional abilities. It consists of three factors: attention to feelings, emotional clarity and mood repair. Participants used a 5-point Likert scale ranging from 1 (strongly disagree) to 5 (strongly agree). Higher scores indicate a stronger agreement with the factor.

Socio-cultural adjustment. The 29 item Socio-Cultural Adaptation Scale (SCAS) developed by Ward and Kennedy [17] was used. Both behavioral and cognitive dimensions were included. Participants answered a 5-point scale ranging from 1 (no difficulty) to 5 (extreme difficulty). Higher scores indicate more adjustment problems. The scale was translated into Spanish and translated back into English by two native English speakers.

Perception of one’s general situation. The perceived general differences between countries were measured through one question. We asked participants if regarding their general situation, and not only the socioeconomic sphere, they were currently worse off (1), the same (2), or better off (3) in Spain than in their home country.

## 3. Results

Attrition analyses were performed to determine whether participants included in this study (those who have participated both times, *n* = 63) differed from the dropouts (*n* = 75) with respect to their baseline levels on the study’s variables. *T*-test and cross tabulation results showed that the two samples did not differ regarding age (*t*_(137)_ = −0.79, *p* = 0.43); country of origin (*χ*^2^_(138,1)_ = 3.04, *p* = 0.08); perceived health (*t*_(137)_ = −1.87, *p* = 0.06); or EI: attention to feelings (*t*_(137)_ = 1.24, *p* = 0.22), clarity in feelings (*t*_(137)_ = 1.04, *p* = 0.30), and mood repair (*t*_(137)_ = −0.32, *p* = 0.75). There were also no differences in socio-cultural adjustment, both in its behavioral (*t*_(137)_ = 1.55, *p* = 0.12) and cognitive (*t*_(137)_ = 0.82, *p* = 0.42) dimensions, or perceptions about differences in their general situation (t_(137)_ = 0.79, *p* = 0.43) and psychological distress (*t*_(137)_ = 1.22, *p* = 0.22).

The only sociodemographic difference between both groups was sex, (*χ*^2^_(138,1)_ = 4.37, *p* = 0.037) showing that, while there are almost the same number of women (*n* = 38) as men (*n* = 37) who did not answer in T2, those who did answer the follow up were unequally represented, with nearly double the number of women (*n* = 43) compared to men (*n* = 20). In T1, the percentage of female/male respondents was 58.7% to 41.3%, respectively. In T2, this percentage was 68.25% versus 31.75%. Therefore, following the recommendations of MacKinnon, et al. [41], we used sex as a control variable in this study. In addition, origin was additionally added to guarantee the independence of the variables.

A series of descriptive analyses for each scale and their intercorrelations were performed, followed by a Student’s *t*-test comparing two independent means and analyzing if there were significant differences between males and females and participants from Ecuador and Romania. Consequently, another *t*-test for two dependent means was performed to look into response changes in T1 and T2 on perceived psychological distress, perceived health, EI, socio-cultural adjustment, and the perceived general situation. We then performed a structural equation model (SEM) using the AMOS software package [42]. The absolute goodness-of-fit indexes were the *χ*^2^ goodness-of-fit statistic and the Root Mean Square Error of Approximation (RMSEA). Additionally, we computed the relative index comparative Fit Index (CFI). Values with scores of 0.90 or higher (for CFI) and of 0.08 or lower (for RMSEA) present acceptable fit [43].

In order to test whether the common method variance bias is a problem, we performed Harman’s single factor test using Confirmatory Factor Analysis [44].

### 3.1. Descriptive Analyses

Results reveal that a single factor could not account for the variance in the data [T1 Delta *χ*^2^_(8)_ = 35.84, *p* < 0.001; T2 Delta *χ*^2^_(6)_ = 313.17, *p* < 0.001]. Consequently, our dataset presents no problems in terms of common method variance in both waves. Internal reliability and all partial correlations when controlling for sex and country of origin are presented in Table 1.

Results show adequate reliability in all variables (Table 1). Attention, clarity and repair correlated among themselves significantly both in T1 (*r* ranged from 0.29 to 0.51) and T2 (*r* ranged from 0.50 to 0.59).

In addition, in T1 attention correlated significantly with more perceived psychological distress (*r* = 0.28) and more cognitive adjustment problems (*r* = 0.26). Clarity and mood repair presented no significant results.

Behavioral adjustment correlated significantly with cognitive adjustment in both waves (*r* = 0.85 and *r* = 0.84 respectively). Psychological distress correlated with worse cognitive and behavioral adjustment in T1 (behavioral: *r* = 0.45; cognitive; *r* = 0.44) and T2 (behavioral: *r* = 0.28; cognitive: *r* = 0.28), respectively.

Means and standard deviations of the different variables are presented in Table 2. Student’s *t*-test revealed no differences in any of the variables under study.

There are differences in perceived health in T1 by education level. Participants without formal studies (M = 3.67, DT = 1.15) perceived significantly worse health than the participants with primary (M = 2.25, DT = 0.72) and secondary studies (M = 2.24, DT= 0.63), but not with university education (M = 2.33, DT = 0.82) (*f*_(63)_ = 5.74, *p* = 0.016). In addition, there are significant differences in behavioral adjustment between participants without studies (M = 2.25, DT = 0.86) and those with completed secondary studies (M = 1.75, DT = 0.65) (*f*_(63)_ = 4.24, *p* = 0.047) in T1, and without (M = 3.27, DT = 1.93) and with university studies in T2 (M = 1.84, DT = 1.61) (*f*_(63)_ =2.81, *p* = 0.047). Distress in T2 also differs in relation to educational level. Participants without formal education (M = 20.50, DT = 4.95) showed a higher level of perceived distress than migrants who had completed university studies (M = 13.50, DT = 6.66) (*f*_(63)_ = 2.98, *p* = 0.039).

Regarding country of origin, differences were found in behavioral adjustment in T1 (*f*_(63)_ = 10.11, *p* = 0.002) and T2 (*f*_(63)_ = 6.76, *p* = 0.002), and in cognitive adjustment in T1 (*f*_(63)_ = 8.23, *p* = 0.006) and T2 (*f*_(63)_ = 5.84, *p* = 0.019). In all cases, participants from Ecuador showed worse socio-cultural adjustment. Differences were also found in attention to feelings in T1 (*f*_(63)_ = 7.69, *p* = 0.007) but not in T2 (*f*_(63)_ = 0.7, *p* = 0.793). Participants from Ecuador showed higher levels than Romanians, indicating that they tended to monitor more one’s own moods and emotions.

Finally, no significant differences were found between answers given in T1 and T2 on any of the variables. An analysis (data not presented) comparing those participants with a longer stay in Spain (75 percentile: 9 years), and those with the shortest stay (25 percentile: 5 years) showed that length of stay did not play a role in changing patterns of sociocultural adaptation, perceived psychological distress, perceived health, EI and the perceived general situation.

### 3.2. Model Testing

**Figure 1:**
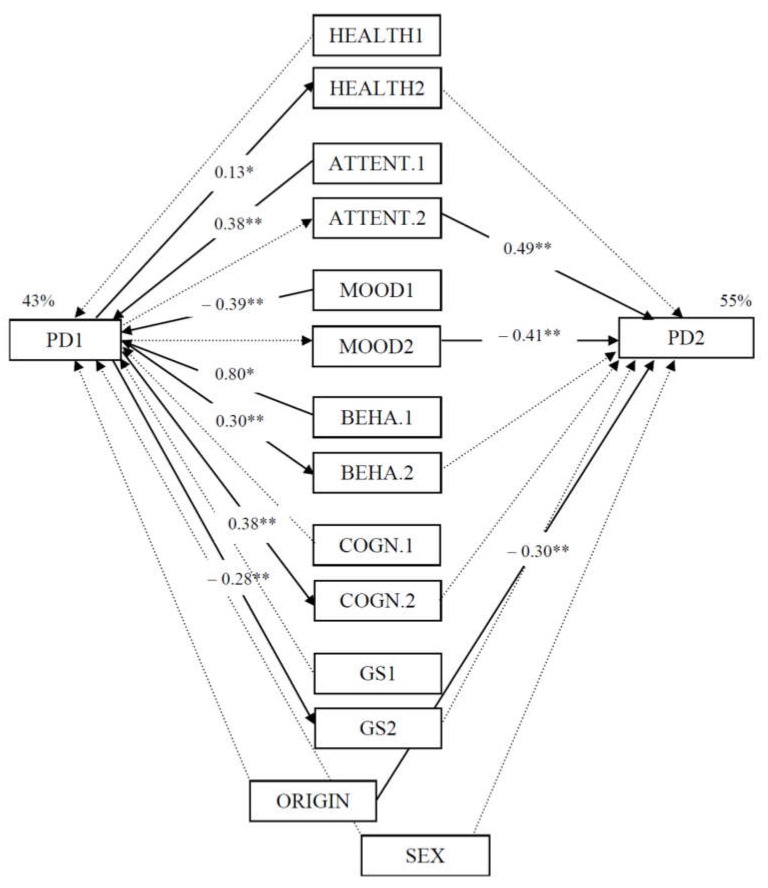
The research model with standardized paths coefficients. PD (psychological distress), HEALTH (perceived health), ATTENT. (Attention to feelings), MOOD (Mood repair), BEHA. (socio-cultural adjustment, behavioral dimension), COGN. (socio-cultural adjustment, cognitive dimension), GS (general situation). ** *p* ≤ 0.01, * *p* ≤ 0.05.

An SEM using the Maximum Likelihood Estimation method was used. The model is presented in Figure 1 showing good fit (*χ*^2^ = 157.48; RMSEA = 0.06; CFI = 0.91). Sex and country of origin were defined as covariates due to the minor differences found in previous comparisons.

The model presented is composed only by statistically significant paths. For this reason, the clarity dimension has been removed from both waves. Results show that perceived psychological distress in T1 is affected by both dimensions of EI (attention to feelings and mood repair), and the behavioral dimension of socio-cultural adjustment in T1. The lower the attention to feelings, the higher the mood repair, and higher the behavioral adjustment in T1, the less the perceived psychological distress. Perceived psychological distress in T1 had an effect on perceived health, on both dimensions of socio-cultural adjustment and on the perception of the general situation in T2. Less perceived psychological distress in T1 triggered better perceived health and better socio-cultural adjustment in T2, and also the perception of a better general situation in Spain compared with their home country.

Moreover, both dimensions of EI in T2 had an effect on perceived psychological distress in T2. The greater the attention to feelings, the more the perceived psychological distress, and more mood repair implied less perceived psychological distress.

With regard to country of origin, migrants from Ecuador, in comparison to those from Romania, stated a higher level of perceived psychological distress in T2. This result is consistent with the higher level of cognitive and behavioral adjustment problems mentioned by these same participants.

## 4. Discussion

There is stability of results and scores after a 13–16 month period regarding psychological distress, adjustment and perception of one’s own situation. These results support those presented by Jasinskaja-Lahti, Horenczyk, and Kinunen [45], stating that the psychological reaction to migration and acculturation does not last longer than 3–5 years. There is an increase in stress levels until the third year after arrival, followed by a decline and a subsequent return to normal levels [46]. The results from our study attest to the fact that length of stay may play a diminishing role in explaining acculturation and psychological distress after a certain period of time in both groups of migrants.

EI and sociocultural adjustment have an impact on perceived psychological distress, confirming our first hypothesis. Whilst clarity is not related to mental well-being, repair is a positive predictor of mental well-being. This result is in line with previous data [47] which show that those individuals who believe that they can regulate their negative mood states direct their attention resources towards coping and maintain a positive outlook, suffer less the impact of stressful events. These results are also consistent with [48], who found that there were no significant differences in clarity scores in a sample of generalized anxiety disorder patients in comparison to non-patients. Previous studies [49,50] have stated that being able to interrupt and regulate negative emotional states and extend positive ones facilitates coping with stressful situations. Other models testing how distress could be associated with T1 and T2 variables, or if distress would predict variables in T1 and T2, were also tested, but results supported the model presented in the study (data not shown but available from authors).

In line with hypothesis 2, whilst being able to regulate negative moods and prolong positive ones is linked to better perceived mental well-being, focusing on one’s mood and emotions is an important predictor of negative mental well-being. Paying too much attention towards emotion is linked to ruminative strategies (guilt), avoidance, loss of control, and health problems [51]. Focusing attention on emotions has been found to be related to maladaptive stress coping strategies based on thought suppression, avoidance, rumination and self-blame [52]. Naming and thinking about one’s emotions are not necessarily positive ways to cope with psychological distress and cognitive problems if one does not have sufficient abilities or resources to repair negative moods in everyday life and interactions [48]. The results from this study coincide with Baudry et al.’s [23] assertion that improving all emotional competences is not necessarily positive. Interventions should focus on the interplay of individual and contextual factors to understand which competences are more suited in a particular situation. It is important to stress in health care interventions that just thinking about one’s emotions regarding the migration experience will not necessarily lead to more positive mental health or well-being but, in fact, could direct towards rumination, stronger emotional intensity and the use of more dysfunctional coping mechanisms. Fernández-Abascal and Martín-Díaz [53] point out that participants who scored high on the attention domain were those who reflected more anxiety and discomfort when observing other people’s negative experiences, situations that can frequently appear in interactions in new cultures different from one’s own due to the need to learn new ways of interacting with contexts in which one’s previous interaction processes may diverge from those of the host majority population.

This is an interesting result for migrant populations that may have to ruminate or avoid their feelings in order to understand new situations and environments. Learning how to adequately regulate emotions (on the basis of both individual needs and social constrains) is a positive well-being factor which should be included by professional practitioners in health promotion programs to avoid enhancing negative outcomes [54].

Country differences in attention to one’s mood and emotions may be explained by the higher collectivistic scores found in Ecuador in comparison to Romania [55]. Collectivism implies adjusting one’s behaviors to the group or to a particular context. This leads to a re-elaboration and suppression of thoughts and feelings which may conflict with the existing social order. As Matsumoto et al. [56] state, this has positive social order consequences, satisfying group needs because it maintains close relations and hierarchy. Nevertheless, on an individual level, if attention derives from suppression as a way of regulating emotion, this can lead to less happiness and stronger maladjustment. As we have seen, attention is positively correlated in both T1 and T2 with mood repair, stressing that the more participants think about their feelings, the more they regulate their moods. Inhibition of feelings and suppression of expression are dysfunctional forms of emotional regulation negatively associated with psychological well-being [57]. Interventions should bear in mind the cultural differences in emotional expression and explain the possible consequences of these differences in new social interactions and environments.

Psychological distress plays an important role in health and psychological adjustment more than a year later. Those participants who stated they were more distressed were also those who afterwards would state that their general health was worse and that they were in general, not only socioeconomically, worse off in Spain in comparison to their home country. These results confirm that positive mental well-being has implications in the long term, and that creating positive contexts for migrants to interact with and manage their lives (i.e., by explaining cultural differences and the importance of emotional regulation) may have a lasting and positive effect on mental well-being.

## 5. Conclusion

### 5.1. Limitations

First, the number of participants is scarce; although most participants stated that they would have answered the follow up study if still living in the city, due to the nature of the labor market and its need for mobility many participants had moved to another city or were out of town for long periods of time. Nevertheless, attrition results show that participants who answered in both waves were not significantly different in their responses to those who answered only in T1. In fact, the only statistical difference in attrition rate was between men and women. As in other studies conducted with small sample sizes using either a qualitative or quantitative methodology, results must be interpreted with caution so as to not overextend and generalize conclusions to other social groups or situations. Although well designed research with a small sample size can yield important results it may also produce type II errors by interpreting as non-significant results which truly are significant. Nevertheless, various authors [58,59] have stated that finding a significant result with small samples could imply that the effect must be large enough to detect it with a small number of participants and as such is an important finding. The fact that our results are robust and in accordance with a lengthy theoretical background in the field leads us to contend that our results are consistent and an addition to the scarce literature. Nonetheless, a large sample size (including participants from more countries) in future studies will allow results to be a more precise estimate of the studied effect and make it easier to access the representativeness of the sample and generalize results [36].

Second, this research is based upon follow-up and self-reported data recorded over a relatively short time span. As can be the case in general self-reports, results could be influenced by social desirability responding, although this is not necessarily always something negative [60]. The results of this study urge researchers to start using multiple waves of data collection over a longer period of time in the analysis of migrant adaptation.

Third, only two different migrant groups were included in the study. Moreover, we could not conduct separate SEMs to test for group differences. Nevertheless, there were no relevant sex or country of origin differences in any variables under consideration. Although the sample size is small, and we must be cautious with the over-generalization of results, controlling the sex and country of origin allows us to stress the validity of the results, their alignment with other emotion related studies, and their importance as a novel, and overlooked, approach to the analysis of acculturation well-being and public health issues over time.

### 5.2. Main Contributions

Due to the lack of published studies on the focus of this research, this study can be an addition to the scarce literature on acculturation, emotions and mental well-being and health for a number of reasons. The results of this study can lead to a series of recommendations in health programs and interventions with migrant populations similar to those studied in this research. First, it is necessary to develop longitudinal or follow-up studies that allow us to analyze the relationship between migration and mental well-being as a process. Research designs which aim to inform public health policies should stress the longitudinal analyses of variables and the nature of possible changes. Furthermore, the results from this study could be applied within a public health context. The training of migrants in EI can be beneficial and necessary as it will help them overcome possible social obstacles and present less psychological and cultural distress, as noted in a recent study [61]. Moreover, practitioners should be aware that the inclusion of emotion-related programs in their interventions with migrants may help improve the mental well-being of this population. These interventions should allow participants to regulate moods and not only base efforts on thinking about one’s feelings.

A second important aspect of this study is the inclusion of variables neglected in most previous studies. The analysis of how emotions and their regulation have a significant impact on migrants’ health has important consequences in the development of programs aimed towards reducing mental distress. Adjustment to one’s new culture, both in terms of learning new cultural codes (cognitive and emotional) and learning how to act in different situations, is an important factor in well-being. In stressful situations, people tend to pay excessive attention to their negative emotional states which, in turn, can lead to more distress. Hampton, Peter, Corus and Brinberg [62] mentioned that emotions play a vital role in health decision strategies, and that cognitive-based interventions may not address all the relevant variables in health programs. Interventions and counseling which focus too much on one’s feelings may not be beneficial for migrants if other measures, such as learning how to repair negative emotional experiences, are not implemented at the same time (e.g., distraction, increasing social support, retrieving positive memories). Interventions must clearly state that paying attention to one’s emotions is positive if one has the possibility of regulating the expression of these emotions, planning actions to improve one’s mood, or reevaluating the situation stressing the positive aspects of migration and distancing oneself from the negative consequences of this migration experience [63,64]. As Baudry et al. state [23], it is important to understand the impact of how these emotions are used over time to focus interventions and public health programs on specific domains (e.g., personal relationships, response to stressful situations, community interactions and social bonds) that may help migrants improve their ability to manage their new environment in different settings.

Third, although the study comprises a relatively small sample, the strength of the paths in the SEM, as well as its theoretical background, and the alignment of results with previous studies conducted with different populations, point to the relevance of including emotion-related variables in the explanation of migrants’ mental well-being due to the persistent impact of these factors. Looking after migrants’ health is cost effective, facilitates their social and personal adaptation, and enhances more adequate public health interventions. As a result, studying and improving the health of all individuals and collectives within society should be an aim of research and interventions conducted within the social and medical sciences.

## Figures and Tables

**Table 1:** Internal consistencies and partial correlations between perceived distress, perceived health, emotional intelligence (EI), socio-cultural adjustment and general situation across time in Ecuadorian and Romanian migrants.

	α	2	3	4	5	6	7	8	9	10	11	12	13	14	15	16
1. Psy. Dist. T1	0.77	0.44 ***	0.24 *	0.12	0.24 *	−0.02	−0.28*	−0.13	−0.26	0.02	0.43 ***	0.19	0.44 **	0.24	−0.17	−0.26 *
2. Psy. Dist. T2	0.88		0.26 *	0.01	0.32 *	0.25	−0.01	−0.02	0.02	−0.15	0.44 ***	0.27 *	0.29 *	0.28 *	−0.04	−0.41 **
3. Perc. heal. T1				0.60 ***	−0.07	0.27 *	−0.26	0.20	−0.04	0.22	0.35 **	0.01	0.32 *	0.03	0.16	−0.03
4. Perc. heal. T2					0.03	0.07	−0.08	0.04	−0.01	0.09	0.02	0.05	0.03	0.06	0.06	0.04
5. Attention T1	0.87					0.25	0.26 *	−0.13	0.25 *	−0.10	0.21	0.27 *	0.27 *	0.33 **	−0.08	−0.08
6. Attention T2	0.84						−0.19	0.50 ***	0.06	0.54 ***	0.20	0.15	0.13	0.16	−0.07	−0.02
7. Clarity T1	0.79							0.07	0.49 ***	−0.18	−0.09	0.06	−0.07	0.01	−0.05	0.08
8. Clarity T2	0.87								0.08	0.59 ***	0.06	0.08	0.12	0.06	−0.04	0.06
9. Mood rep. T1	0.93									0.17	0.03	0.12	0.07	0.16	0.15	0.24
10. Mood rep. T2	0.90										0.15	0.11	0.18	0.23	−0.03	0.23
11. Beh. adj. T1	0.92											0.46 ***	0.86 ***	0.44 **	0.21	−0.29 *
12. Beh. adj.T2	0.87												0.35 **	0.85 ***	0.13	−0.08
13. Cog. adj. T1	0.94													0.45 **	0.12	−0.16
14. Cog. adj.T2	0.87														−0.45	−0.04
15. Gen. sit. T1																0.16
16. Gen. sit. T2																

Note. Controlling for socio-economic status, sex and country of origin. N = 63. *** *p* ≤ 0.001, ** *p* ≤ 0.01, * *p* ≤ 0.05.

**Table 2:** Means and standard deviations of the variables of interest.

Variables	Sex				Origin				Wave			
	Male		Female		Romanian		Ecuadorian		Time 1	*SD*	Time 2	*SD*
	*M*	*SD*	*M*	*SD*	*M*	*SD*	*M*	*SD*				
Psychological Distress T1	9.81	5.21	11.09	4.87	9.90	4.73	11.07	5.11	10.68	4.99		
Psychological Distress T2	10.15	6.64	12.40	6.58	12.95	7.29	11.05	6.26			11.68	6.63
Perceived health T1	2.16	0.69	2.40	0.79	2.52	0.60	2.22	0.82	2.32	0.76		
Perceived health T2	2.25	0.85	2.37	0.73	2.52	0.60	2.24	0.82			2.33	0.76
Attention T1	3.28	0.86	3.31	0.94	2.88	0.97	3.52	0.80	3.31	0.91		
Attention T2	3.38	0.67	3.21	1.02	3.31	0.92	3.24	0.93			3.27	0.92
Clarity T1	3.40	0.69	3.47	0.87	3.27	0.88	3.54	0.77	3.45	0.81		
Clarity T2	3.82	0.82	3.40	0.99	3.74	0.89	3.43	0.99			3.54	0.95
Mood repair T1	3.96	0.76	4.04	0.63	3.80	0.64	4.12	0.66	4.02	0.66		
Mood repair T2	4.13	0.64	3.80	0.82	3.92	0.68	3.90	0.83			3.91	0.78
Behavioral adjustment T1	2.01	0.64	1.98	0.79	1.60	0.67	2.19	0.70	1.99	0.74		
Behavioral adjustment T2	2.10	0.90	1.80	0.66	1.56	0.45	2.06	0.81			1.89	0.74
Cognitive adjustment T1	2.18	0.87	2.18	0.96	1.73	0.87	2.41	0.88	1.90	0.75		
Cognitive adjustment T2	2.19	0.90	1.93	0.78	1.67	0.67	2.19	0.85			2.01	0.82
General situation T1	2.55	0.61	2.49	0.75	2.57	0.68	2.48	0.72	2.51	0.70		
General situation T2	2.70	0.47	2.53	0.74	2.62	0.59	2.57	0.70			2.59	0.60

Note. N = 63. Male’s *n* = 20; Female’s *n* = 43; Romanian’s *n* = 21; Ecuadorian’s *n* = 42.
